# Identification of Key Molecular Pathways and Associated Genes as Targets to Overcome Radiotherapy Resistance Using a Combination of Radiotherapy and Immunotherapy in Glioma Patients

**DOI:** 10.3390/ijms25053076

**Published:** 2024-03-06

**Authors:** Tianqi Zhang, Qiao Zhang, Xinwei He, Yuting Lu, Andrew Shao, Xiaoqiang Sun, Yongzhao Shao

**Affiliations:** 1 Department of Population Health, New York University Grossman School of Medicine, New York, NY 10016, USA; tianqi.zhang@nyulangone.org (T.Z.); qiao.zhang@nyulangone.org (Q.Z.); yl4036@nyu.edu (Y.L.); 2 School of Mathematics, Sun Yat-sen University, Guangzhou 510275, China; hexw26@mail.sysu.edu.cn; 3 Center of Data Science, New York University, New York, NY 10011, USA; as13381@nyu.edu

**Keywords:** tumor-associated macrophage cells, tumor microenvironment, translational research strategy, radiation therapy, immunotherapy, CSF-1R

## Abstract

Recent mechanistic studies have indicated that combinations of radiotherapy (RT) plus immunotherapy (via CSF-1R inhibition) can serve as a strategy to overcome RT resistance and improve the survival of glioma mice. Given the high mortality rate for glioma, including low-grade glioma (LGG) patients, it is of critical importance to investigate the mechanism of the combination of RT and immunotherapy and further translate the mechanism from mouse studies to improve survival of RT-treated human glioma patients. Using the RNA-seq data from a glioma mouse study, 874 differentially expressed genes (DEGs) between the group of RT-treated mice at glioma recurrence and the group of mice with combination treatment (RT plus CSF-1R inhibition) were translated to the human genome to identify significant molecular pathways using the KEGG enrichment analysis. The enrichment analysis yields statistically significant signaling pathways, including the phosphoinositide 3-kinase (PI3K)/AKT pathway, Hippo pathway, and Notch pathway. Within each pathway, a candidate gene set was selected by Cox regression models as genetic biomarkers for resistance to RT and response to the combination of RT plus immunotherapies. Each Cox model is trained using a cohort of 295 RT-treated LGG patients from The Cancer Genome Atlas (TCGA) database and validated using a cohort of 127 RT-treated LGG patients from the Chinese Glioma Genome Atlas (CGGA) database. A four-DEG signature (ITGB8, COL9A3, TGFB2, JAG1) was identified from the significant genes within the three pathways and yielded the area under time-dependent ROC curve AUC = 0.86 for 5-year survival in the validation set, which indicates that the selected DEGs have strong prognostic value and are potential intervention targets for combination therapies. These findings may facilitate future trial designs for developing combination therapies for glioma patients.

## 1. Introduction

Despite the impressive advances of cancer immunotherapy via immune checkpoint inhibitors (ICIs) in treating many types of cancer in the last decade, diffuse low-grade glioma (LGG) (grade II/III) is still largely incurable, which induces profound disability and high mortality. More than half of LGGs evolve and progress to grade IV glioma (glioblastoma multiforme, GBM), which has a dreadful prognosis with a median survival of less than two years.

A leading hypothesis for the lack of efficacy of ICI-based immunotherapies in diffuse gliomas is that the commonly used ICIs may have focused on the wrong targets in gliomas. As is well known, the commonly used ICIs such as nivolumab, pembrolizumab, and ipilimumab all reduce immune suppression mediated by regulatory T-cells (Tregs) through blockade of either the PD1/PD-L1 axis or the CTLA4. However, there is strong evidence indicating that immune suppression in gliomas is predominantly performed by tumor-associated macrophages (TAMs), not Tregs. It is known that macrophages can be polarized toward a pro-inflammatory/antitumor phenotype (M1) or an anti-inflammatory/immune suppressive phenotype (M2). Gliomas and other cancers have been shown to have the capacity for polarizing macrophages toward the pro-tumor M2 phenotype. In particular, the colony-stimulating factor-1 receptor (CSF-1R or CD115) plays an important role in macrophage development and polarization. Specifically, binding of CSF-1 to CSF-1R triggers auto-phosphorylation of the receptor on several tyrosine residues, and this can activate multiple intracellular pathways, including the phosphatidyl inositol 3-kinase (PI3K) pathway, which promotes macrophage maturation and upregulates expression of genes that lead to the pro-tumor M2 phenotype [1]. This leads to great interest in investigating the therapeutic potential of CSF-1R inhibitors in cancer treatment. Indeed, in a glioma mouse study, the inhibition of CSF-1R with BLZ-945 resulted in a reduction of M2 polarization within the tumor microenvironment and decreased tumor growth rates [2]. Nevertheless, a subsequent study indicated that persistent inhibition of CSF-1R alone was inadequate for long-term tumor control due to drug resistance, and glioma growth resumed after an initial period of slow proliferation [3].

Radiotherapy (RT) has been widely used as a fundamental component of cancer treatment received by about half of all patients with cancer, including glioma [4,5]. RT is traditionally delivered for purposes of local control, but many RT-treated cancer patients relapse with local tumor recurrence and distant metastasis. The perception of RT as a simple local treatment for tumors has undergone a significant transformation in recent years. It is now widely acknowledged that RT has the potential to trigger a systemic immune response and reprogram the tumor microenvironment (TME) [6,7,8]. This provides a compelling rationale for combining RT with immunotherapies to develop novel treatments.

Increasing evidence suggests that the treatment combining radiotherapy (RT) with immunotherapy via colony-stimulating factor-1 receptor (CSF-1R) inhibition is promising to improve survival over RT alone or CSF-1R inhibition alone. A promising report showed that the CSF-1R inhibitor enhanced the efficacy of RT and reduced infiltration of myeloid suppressor cells in an orthotopic and heterotopic mouse model using the human GBM cell line U251 [9]. To further understand the dynamics of the combined RT plus CSF-1R inhibition anti-glioma therapy, Akkari et al. (2020) [10] conducted an in-depth investigation of the dynamic changes in different tumor-associated macrophages (TAMs) in a randomized mouse study. Specifically, they explored how RT dynamically influences the relative abundance and phenotypes of brain-resident microglia (MG) and peripherally recruited monocyte-derived macrophages (MDMs) in glioma mice. The study identified radiation-specific, stage-dependent gene expression signatures for MG and MDM in murine gliomas, confirming altered expression of several genes and proteins in Notch and Hippo pathways in recurrent murine gliomas. These researchers observed that targeting these TAM populations using a CSF-1R inhibitor BLZ-945 in combination with RT could enhance the efficacy of RT and significantly improve survival in preclinical glioma models. These important findings unveil the dynamics and adaptability of distinct macrophage populations in the irradiated tumor microenvironment, offering translational potential for enhancing the effectiveness of standard-of-care treatment in gliomas. Further support for the effectiveness of the combination therapy has been provided by another independent mouse study. In fact, using an orthotopic, immunocompetent GBM mouse model, Almahariq et al. (2021) [11] showed that inhibition of CSF-1R with BLZ-945 enhanced the efficacy of RT in the glioma treatment and resulted in significantly improved mouse survival compared to RT alone or CSF-1R inhibition alone in murine gliomas. Significantly, more than 70% of mice in the combination therapy group achieved long-term survival, as reported in the study. In summary, recent preclinical mechanistic studies have indicated that the utilization of CSF-1R inhibition, such as through BLZ-945, as a standalone treatment for gliomas, may not be adequate to achieve a significant improvement in survival. However, combining CSF-1R inhibition with RT may significantly enhance RT-induced antitumor immunity, potentially overcoming RT resistance and resulting in long-term improvement in survival outcomes in murine gliomas.

Given the currently poor overall survival rates in glioma patients, an in-depth investigation of the key molecular pathways and mechanism of RT resistance and the combination therapy (RT plus BLZ-945) in these preclinical studies would be desired. Moreover, the translation of mechanistic findings to prolong the survival of human glioma patients is of great clinical significance. There has been no systematic identification of the key molecular pathways that underlie RT resistance and relevance as targets for combination therapies in humans. Here, we focused on three significant signaling pathways [3,10,12], which might underlie RT resistance and the improved efficacy of the combination therapy. As is well known, human gliomas are much more heterogeneous than the animal models studied. It is thus unclear whether the new mechanistic findings from the mouse studies can be translated successfully to multiple independent cohorts of human glioma patients [13,14]. There is an urgent need to conduct a translational study to investigate existing evidence for the potential effectiveness of combinations of radiotherapy (RT) plus immunotherapy via CSF-1R inhibition on multiple independent cohorts of human glioma patients. Our study focused on low-grade glioma (LGG) (grade II/III) patients since studies aimed at identifying effective biomarkers for RT-treated LGG patient prognosis are still limited [15]. Therefore, based on the pathways and mechanistic study findings in murine gliomas, it is of clinical importance to detect prognostic signatures based on DEGs and associated pathways that underlie the effect of CSF-1R inhibition among RT-treated LGG patients to optimize novel therapeutic strategies.

In this paper, we first identified the key molecular pathways reflecting the mechanisms of RT resistance, and then we evaluated the utility of key genes in these identified pathways as targets for combination therapy using CSF-1R inhibition to prolong the survival of the RT-treated LGG patients. Specifically, borrowing strength from the existing RNA-seq gene expression data from an in-depth mouse study of Akkari et al. (2020) [10], we identified a set of differentially expressed genes (DEGs) between the monotherapy (RT) treated mice and those under the combination treatment (RT plus CSF-1R inhibition). We then translated the DEGs identified from mouse samples into those in the human genome based on the orthology mapping. Subsequently, enrichment analyses were conducted for mouse and human, separately, which identified three significantly enriched pathways, i.e., phosphoinositide 3-kinase (PI3K)/AKT pathway, Hippo pathway, and Notch pathway. Within each pathway, we identified a gene signature using the Cox regression model using RNA-seq data from a cohort of 295 irradiated LGG patients from The Cancer Genome Atlas (TCGA) database on the NCI website. As an independent cohort of the validation set to evaluate the prognostic accuracy of the Cox model based on the DEGs, we used 127 irradiated LGG patients in the Chinese Glioma Genome Atlas (CGGA). Towards this end, time-dependent ROC curves and corresponding AUCs were used to demonstrate the prognostic performance of the identified genetic biomarkers in the irradiated LGG patients and non-irradiated LGG patients, respectively. In addition, Kaplan–Meier (KM) curves were generated and compared between high versus low-risk scores. Finally, we constructed a gene signature by selecting the significant genes from the Cox models built for each of the three pathways. The identified genetic biomarkers showed high AUCs at 2-year, 3-year, and 5-year in the irradiated LGG patients in both the training cohort (TCGA) and the independent validation cohort (CGGA), indicating good predictive performance of the identified genetic signature. Elevated expression levels of the signature DEGs are highly predictive of the poor survival of RT-treated LGG patients. One can potentially lower the expressions of these DEGs using CSF-1R inhibition (e.g., using the antibody BLZ-945) among RT-treated glioma patients to achieve survival advantages, as shown in the mouse studies. Thus, the identified gene signature can potentially be used as new targets to optimize therapeutic strategies. In short, the high-impact genes can serve as druggable targets to develop novel immunotherapies for patients not responsive (or resistant) to the current radiation therapies to prolong the survival of these LGG patients. These results can potentially aid the design of human clinical trials to translate the identified mechanisms of the promising mouse studies into effective novel therapies for glioma patients by combining RT with immunotherapies via CSF-1R inhibition.

## 2. Results

We began with the experimental findings of a preclinical mouse study that revealed CSF-1R inhibition as a useful strategy to overcome radiation resistance in murine gliomas [10]. After downloading the preclinical RNA-seq data from the Gene Expression Omnibus (GEO) website (https://www.ncbi.nlm.nih.gov/geo/) with accession number GSE99537 (accessed on 30 May 2023), we selected mouse samples that undertook combination treatment (i.e., radiation plus CSF-1R inhibition) or monotherapy of radiation alone. Here, the selected mouse samples were all reported to develop resistance to radiation; however, combining CSF-1R blockade with radiotherapy was found to yield substantial improvements in overall survival in preclinical models [10]. Based on the promising findings in mice, our objective is to identify the key molecular pathways and associated predictive genes that underlie the mechanism of the combination therapy in overcoming RT resistance in glioma mice. We then would translate these newly identified mechanistic insights from preclinical studies to human patients for the development of a promising combination of therapies involving radiation (RT) and immunotherapies, with the goal of prolonging the survival of glioma patients.

**Figure 1:**
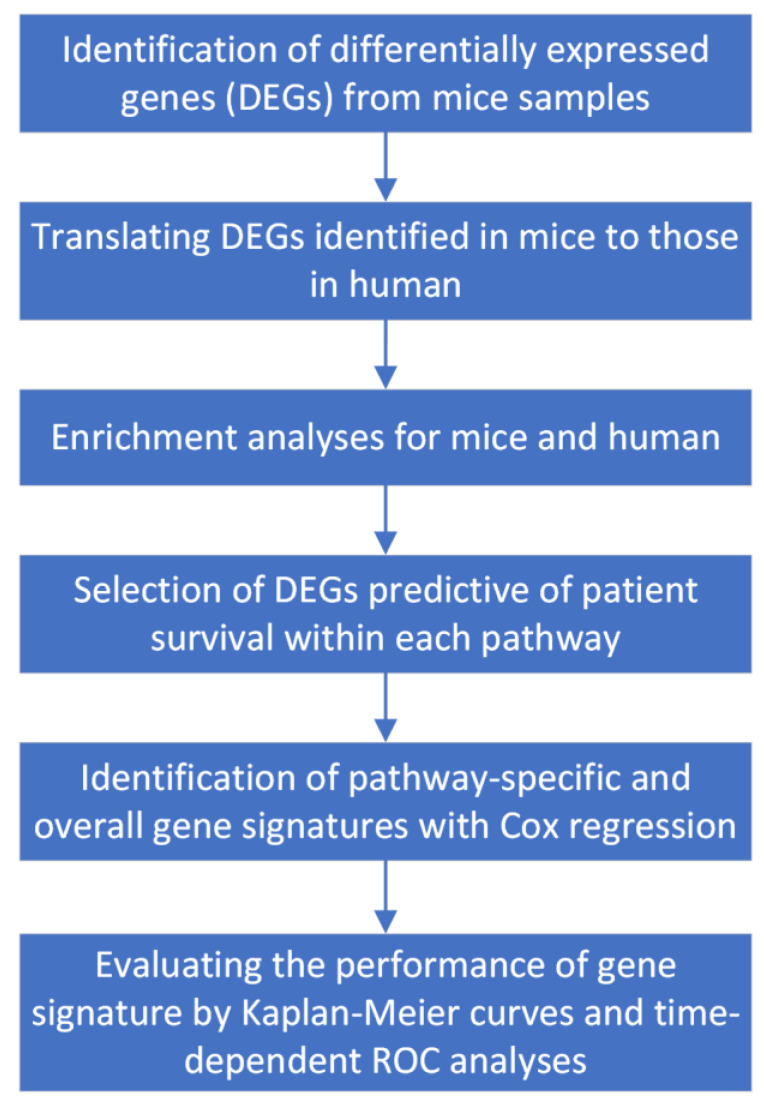
The flow chart of our translational research strategy.

For this purpose, two human glioma datasets (TCGA and CGGA) were used. Since the selected mice took either RT-only or RT plus CSF-1R inhibition, our translation would be mainly focused on the group of irradiated LGG patients. Figure 1 describes the workflow of our translational research strategy applied in this study.

### 2.1. Differentially Expressed Genes (DEG) Analysis in Mouse Data

As described in Section 4.2, we identified the differentially expressed genes (DEGs) between the monotherapy (RT) and combination treatment groups in mice data using DESeq2 [16]. Here, we used the Wald test and mean fit type in DESeq2, such that the significance thresholds were set as *p*-value < 0.05 and log2FoldChange > 0.5. Then, 285 DEGs were identified. The union of the 285 DEGs and the 693 significantly upregulated genes in both MDM and MG from [10] yielded a total of 874 DEGs in mouse, which were translated to human for further analysis based on the orthology in both human and mouse.

### 2.2. Identification of Key Pathways via Enrichment Analysis in Mouse and Human

**Figure 2:**
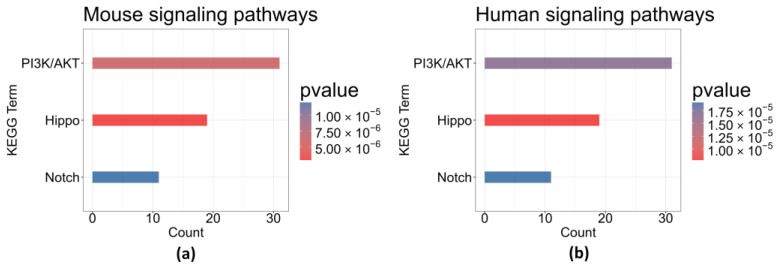
KEGG enrichment analysis of DEGs for signaling pathways in mouse (**a**) and human (**b**).

To gain insight into molecular mechanisms, we performed the enrichment analysis based on the 874 DEGs for mouse and human using the KEGG database separately. The most statistically significant signaling pathway in the KEGG enrichment analysis is the PI3K/AKT pathway, which is of critical importance in CSF-1R inhibition or radiation therapy in mouse glioma models [3,12]. The important roles of multiple DEGs in the Notch and Hippo pathways were also extensively discussed by Akkari et al. (2020) [10]. Here, all three target pathways were found statistically significant in the enrichment analysis, i.e., PI3K/AKT pathway, Hippo pathway, and Notch pathway. The KEGG enrichment bar plots are given in Figure 2. The results indicated that combination therapy of RT with CSF-1R inhibition therapy can target and stabilize these pathways, which were elevated in the RT-only therapy and should reflect the underlying mechanism leading to improved survival of glioma mice and human patients. We identified the pathway-related genes for further analysis. The identified genes for each pathway are given in Table S1 of the Supplementary Materials.

### 2.3. Selection of Genes Predictive of Patient Survival in Each of the Key Pathways

First, the univariate Cox regression model was fitted for each gene in the pathway-related gene set individually using the TCGA cohort of LGG patients treated with radiation therapy. The candidate genes (DEGs) for multivariable Cox model analysis were selected from the univariate Cox regression model if their regression coefficients were positive and *p*-value<0.1. When the number of the candidate genes was large, we performed a Lasso-based Cox regression analysis to shrink the feature set size further. Then, we used the Lasso-selected genes adjusted with clinical information (patient age and glioma grade) to fit a multivariate Cox regression model for each pathway to show the relative strength of each gene. In both the training and validation datasets, a risk score was computed for irradiated LGG patients to arrange samples in descending order. The top 50% of patients were classified as the high-risk group, while the bottom 50% were classified as the low-risk group. For easy visualization of the prognostic utility of the Cox model, we plotted Kaplan–Meier curves comparing the survival of patients with high versus low-risk scores cut at the median risk score. Subsequently, we conducted time-dependent ROC analyses to assess the predictive performance of the selected genes of each pathway. Within each pathway, we also examined the selected genes’ performance in non-irradiated LGG patients. In particular, we refitted Cox regression models with the identified signature in TCGA non-irradiated LGG subjects and re-calculated the risk score based on the refitted model in TCGA non-irradiated LGG (n = 183) and CGGA non-irradiated LGG (n = 36).

#### 2.3.1. Identification and Evaluation of Prognostic Genes in PI3K/AKT Pathway

**Figure 3:**
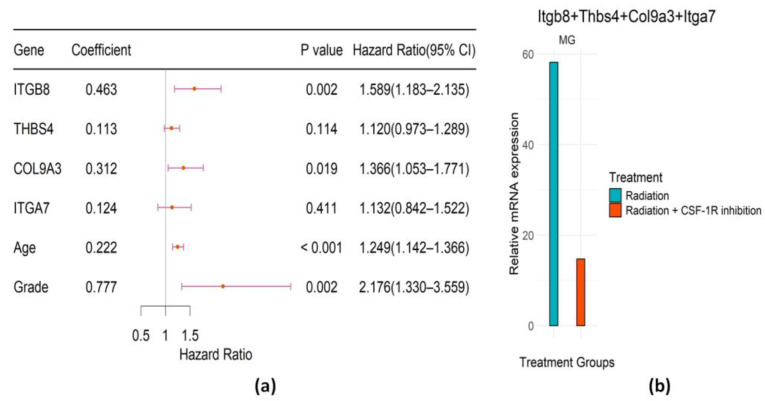
Evaluation of prognostic genes in PI3K/AKT pathway. (**a**) Forest plot of the regression coefficients (log HR) of the Cox PH model, *p*-values, hazard ratios (HRs), and associated 95% confidence intervals. (**b**) Bar plot representing the weighted expression of selected genes in MG in different treatment groups of mice.

With the selected 18 DEGs by the univariate Cox in the PI3K/AKT pathway, we conducted a Cox regression model with the Lasso penalty to shrink the feature set size further. The tuning parameter of the L1-penalty term of the Lasso Cox regression is chosen by 10-fold cross-validation. Then, the genes selected in this pathway were ITGB8, THBS4, COL9A3, and ITGA7. The risk score (RS) formula was obtained from Figure 3a as follows.

(1) RS=0.222∗Age+0.777∗Grade+0.463∗ITGB8+0.113∗THBS4+0.312∗COL9A3+0.124∗ITGA7

The forest plot, together with the coefficient, *p*-value, and hazard ratio of the identified signature in the multivariate Cox regression model, is presented in Figure 3a. The bar plot in Figure 3b represents the expression of selected genes weighted by the Cox regression coefficients in mice preclinical trials. Besides age and grade, ITGB8 and COL9A3 are significant in the multivariate Cox model. The result suggests that the high expression level of these genes is associated with high hazard and poor survival, which is consistent with findings in mice where these genes are all upregulated at glioma recurrence. Therefore, it provides evidence that controlling the up-regulation of these genes in the PI3K/AKT pathway among RT-treated LGG patients using the antibody may have a high likelihood of prolonging their survival. According to the mice mechanistic study, combination therapy of RT with CSF-1R inhibition therapy can reduce the up-regulation of these candidate genes and thus would be hopeful of prolonging patient survival, which can be verified in future clinical trials.

**Figure 4:**
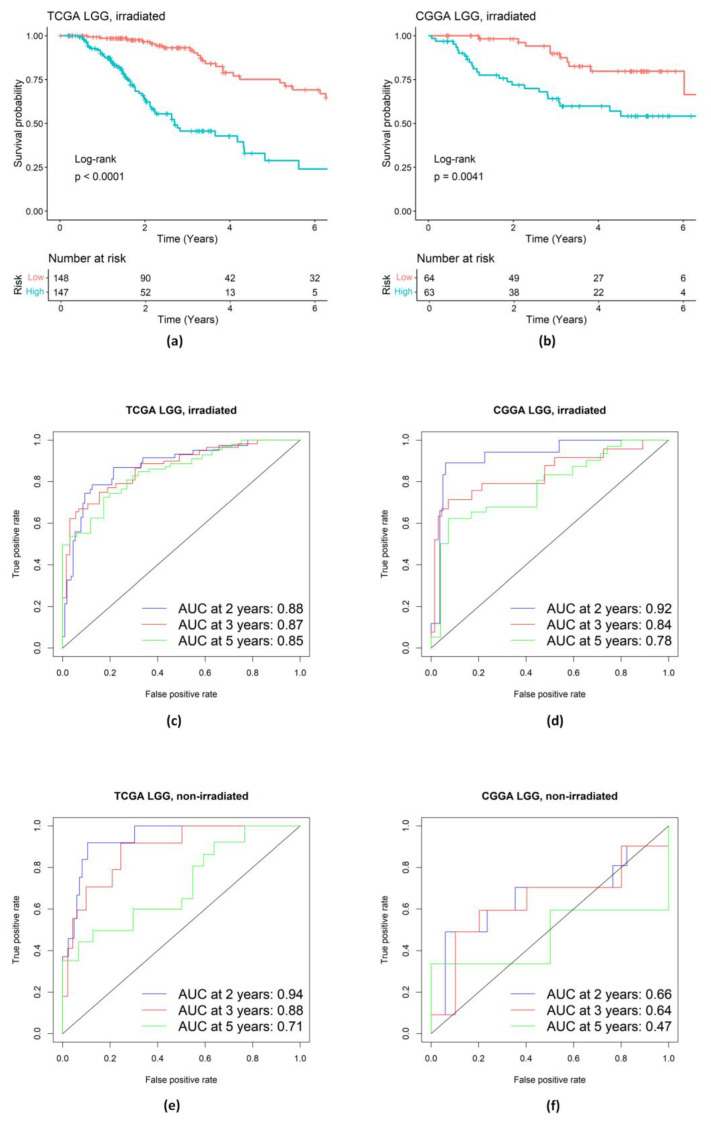
Evaluation of prognostic performance of the genetic signature in PI3K/Akt pathway. (**a**,**b**) KM survival analysis of high- and low-risk groups of LGG patients treated with radiation in the training TCGA dataset and in the validation CGGA dataset. (**c**–**f**) Time-dependent ROC analysis of LGG patients in the training TCGA dataset and in the validation CGGA dataset.

For the irradiated LGG patients, Kaplan–Meier (KM) survival curves for those two groups (high/low risk) of patients were well separated, and the difference was significant according to the log-rank test in both the training and validation datasets (Figure 4a,b). Subsequently, time-dependent ROC analysis was conducted to evaluate prognostic accuracy. For the training dataset of irradiated LGG patients from TCGA, the time-dependent area under the curves (AUCs) for 2-year, 3-year, and 5-year survival rates were 0.88, 0.87, and 0.85, respectively (Figure 4c). In the independent validation dataset from CGGA, the time-dependent AUCs for 2-year, 3-year, and 5-year survival rates were 0.92, 0.84, and 0.78, respectively (Figure 4d). Also, we examined the selected genes for the non-irradiated LGG patients (Figure 4e,f). The results suggest that our identified gene signature in the PI3K/AKT pathway does not work well for non-irradiated LGG patients. For example, the AUC for 5-year survival rates in the non-irradiated CGGA LGG patients (Figure 4f) was only 0.47.

#### 2.3.2. Identification and Evaluation of Prognostic Genes in Hippo Pathway

**Figure 5:**
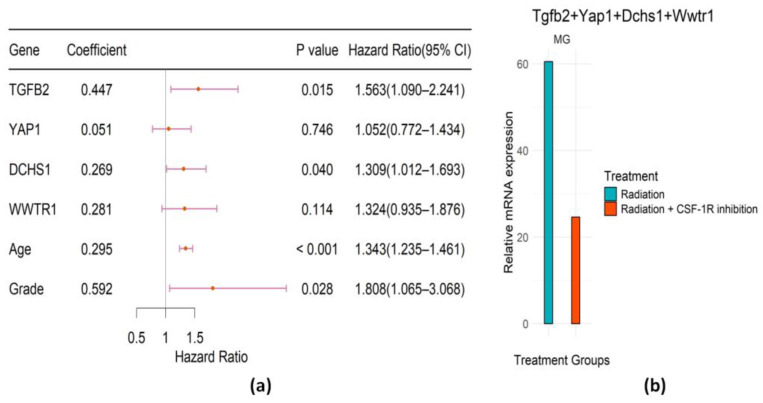
Evaluation of prognostic genes in Hippo pathway. (**a**) Forest plot of the regression coefficients (log HR) of the Cox PH model, *p*-values, hazard ratios (HRs), and associated 95% confidence intervals. (**b**) Bar plot representing the weighted expression of selected genes in MG in different treatment groups of mice.

The identified genes in the Hippo pathway were TGFB2, YAP1, DCHS1, and WWTR1. The forest plot and the bar plot are provided in Figure 5a,b. In Cox regression, TGFB2 and DCHS1 are associated significantly with the survival of irradiated LGG patients. The risk score (RS) formula was obtained from Figure 5a as follows.

(2) RS=0.295∗Age+0.592∗Grade+0.447∗TGFB2+0.051∗YAP1+0.269∗DCHS1+0.281∗WWTR1

These results suggest that combination therapy of RT with CSF-1R inhibition therapy has the potential to reduce the up-regulation of these identified genes in the Hippo pathway and prolong glioma patients’ survival.

**Figure 6:**
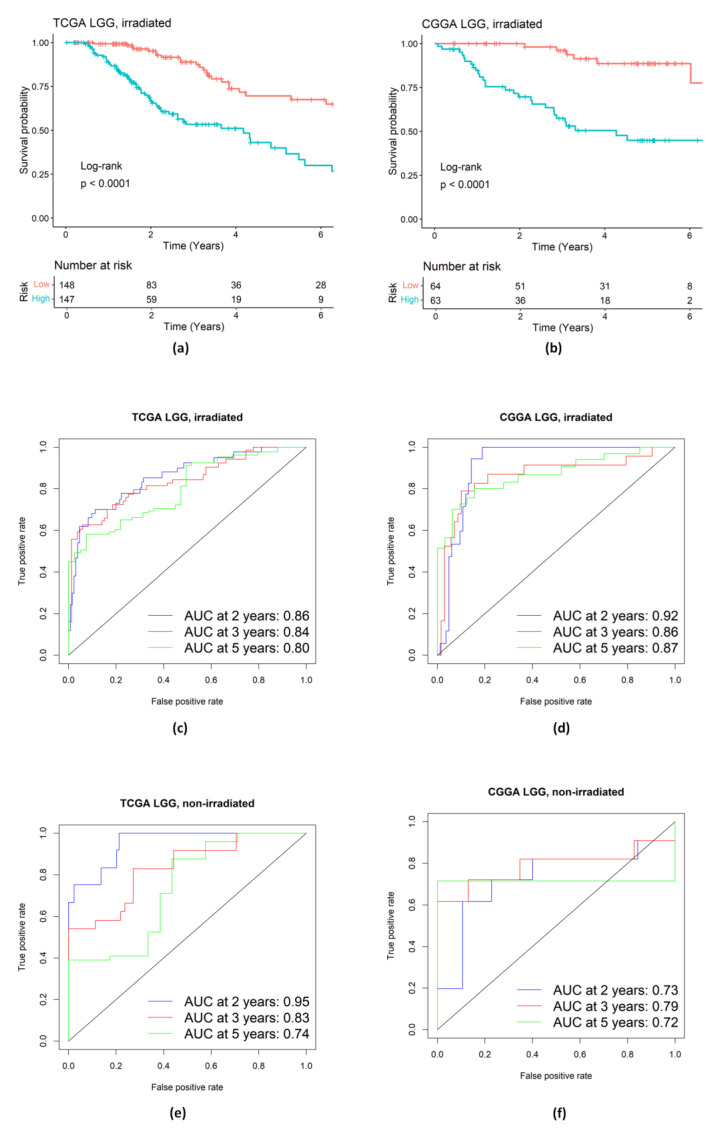
Evaluation of prognostic performance of the genetic signature in Hippo pathway. (**a**,**b**) KM survival analysis of high- and low-risk groups of LGG patients treated with radiation in the training TCGA dataset and in the validation CGGA dataset. (**c**–**f**) Time-dependent ROC analysis of LGG patients in the training TCGA dataset and in the validation CGGA dataset.

With the identified genes in the Hippo pathway, the KM curves (Figure 6a,b) for those two groups (high/low risk) of irradiated LGG patients were well separated, and the difference was significant according to the log-rank test in both the training and validation datasets. We conducted a time-dependent ROC analysis in Figure 6c–f. The results suggest that our identified gene signature in the Hippo pathway works well for the irradiated LGG patients but not for the non-irradiated LGG patients. In particular, for the validation data set of non-irradiated LGG patients (Figure 6f), the AUC at 5 years was 0.72, which was noticeably lower than the corresponding value of 0.87 (Figure 6d) for the validation data set of irradiated LGG patients.

#### 2.3.3. Identification and Evaluation of the Prognostic Genes in Notch Pathway

**Figure 7:**
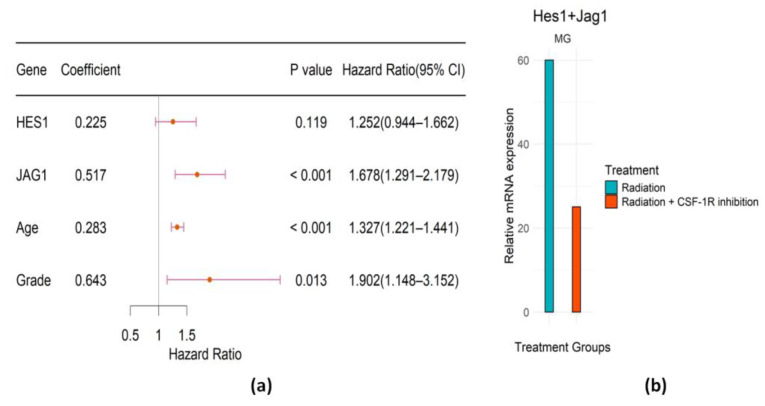
Evaluation of the prognostic genes in Notch pathway. (**a**) Forest plot of the regression coefficients (log HR) of the Cox PH model, *p*-values, hazard ratios (HRs), and associated 95% confidence intervals. (**b**) Bar plot representing the weighted expression of selected genes in MG in different treatment groups of mice.

With the univariate Cox regression model, the selected genes in the Notch pathway are HES1 and JAG1. The risk score (RS) formula was obtained from Figure 7a as follows:

(3) RS=0.283∗Age+0.643∗Grade+0.225∗HES1+0.517∗JAG1

In this pathway, only JAG1 is significant in the multivariate Cox model (Figure 7a). Also, Figure 7b indicates that the identified genes are upregulated in the monotherapy (RT) group of mice, compared with the combination treatment group. These provide compelling evidence that CSF-1R inhibition can serve as an effective treatment for irradiated LGG patients, which potentially targets the Notch pathway.

**Figure 8:**
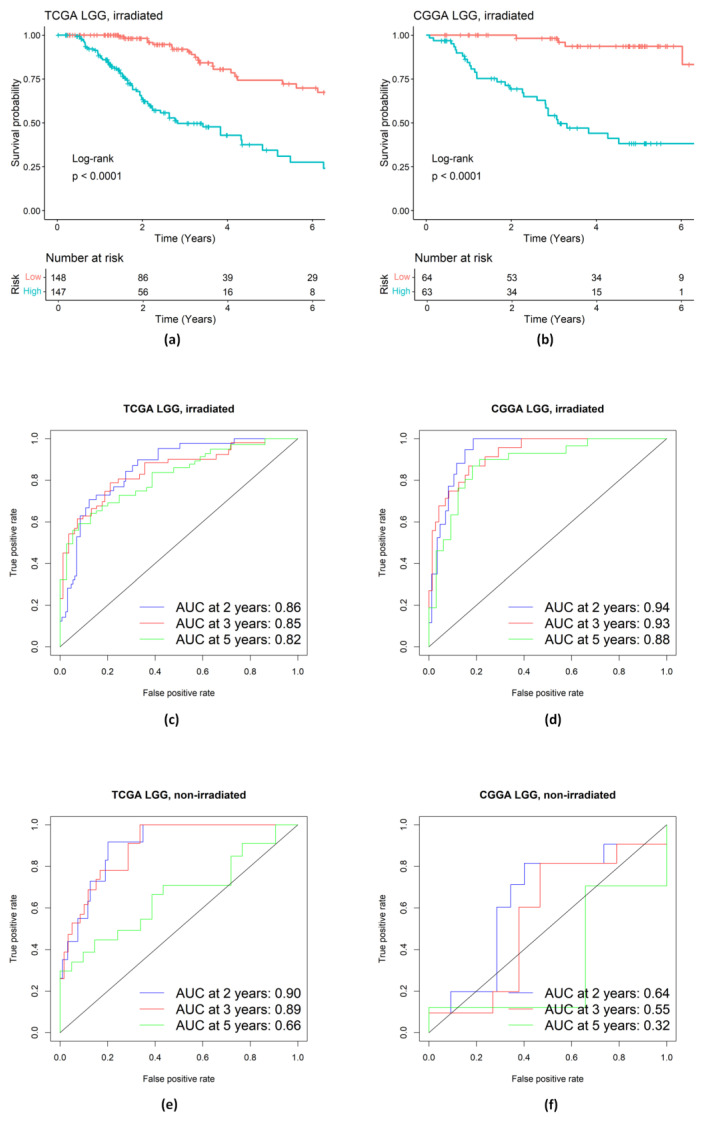
Evaluation of prognostic performance of the genetic signature in Notch pathway. (**a**,**b**) KM survival analysis of high- and low-risk groups of LGG patients treated with radiation in the training TCGA dataset and in the validation CGGA dataset. (**c**–**f**) Time-dependent ROC analysis of LGG patients in the training TCGA dataset and in the validation CGGA dataset.

We conducted Kaplan–Meier analysis and time-dependent ROC analyses to assess the performance of the identified genes in the Notch pathway with respect to predicting patient survival. As shown in Figure 8a–f, the identified signature in this pathway works well for the irradiated LGG patients; however, it still does not work for the non-irradiated LGG patients. For example, the AUC for 5-year survival rates in the non-irradiated LGG patients of CGGA (Figure 8f) was only 0.32.

### 2.4. Predictive Performance of the Identified Significant Genes from Three Pathways

**Figure 9:**
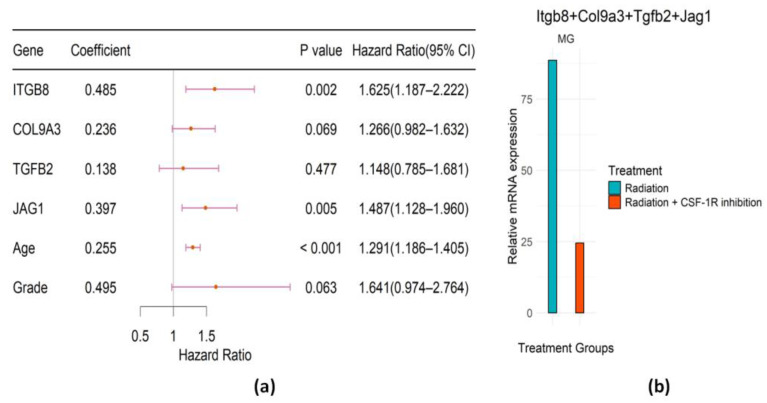
Evaluation of the identified significant genes from the three pathways. (**a**) Forest plot of the regression coefficients (log HR) of the Cox PH model, *p*-values, hazard ratios (HRs), and associated 95% confidence intervals. (**b**) Bar plot representing the weighted expression of selected genes in MG in different treatment groups of mice.

We constructed a gene signature that collected the significant genes (*p*-value<0.02) from the above three pathways, i.e., the PI3K/AKT pathway, Hippo pathway, and Notch pathway. The predictive signature consists of 6 covariates: ITGB8, COL9A3, TGFB2, JAG1, age and grade. The risk score (RS) formula was obtained from Figure 9a as follows:

(4) RS=0.255∗Age+0.495∗Grade+0.485∗ITGB8+0.236∗COL9A3+0.138∗TGFB2+0.397∗JAG1

Figure 9a indicates that a high expression level of these genes is associated with poor survival of irradiated LGG patients, which is consistent with findings in mice studies where these genes are all upregulated in the monotherapy (RT-only) group (Figure 9b). Therefore, it provides evidence that CSF-1R inhibition might target these three pathways to overcome RT resistance, and controlling the up-regulation of these genes among RT-treated LGG patients may have a high likelihood of prolonging their survival. According to the mice mechanistic study, combination therapy of RT with CSF-1R inhibition therapy can reduce the up-regulation of these candidate genes and thus would be hopeful of prolonging patient survival, which can be verified in clinical trials. Importantly, in addition to evidence of up-regulation in mouse mechanistic studies, all these genes have been reported in different studies as being associated with glioma and other cancer progression. More details on these signature genes will be provided in the Discussion. Moreover, we provide the KM analysis and the bar plots that show the expression of each selected gene in MG in different treatment groups of mice in Figure S1 of the Supplementary Materials.

**Figure 10:**
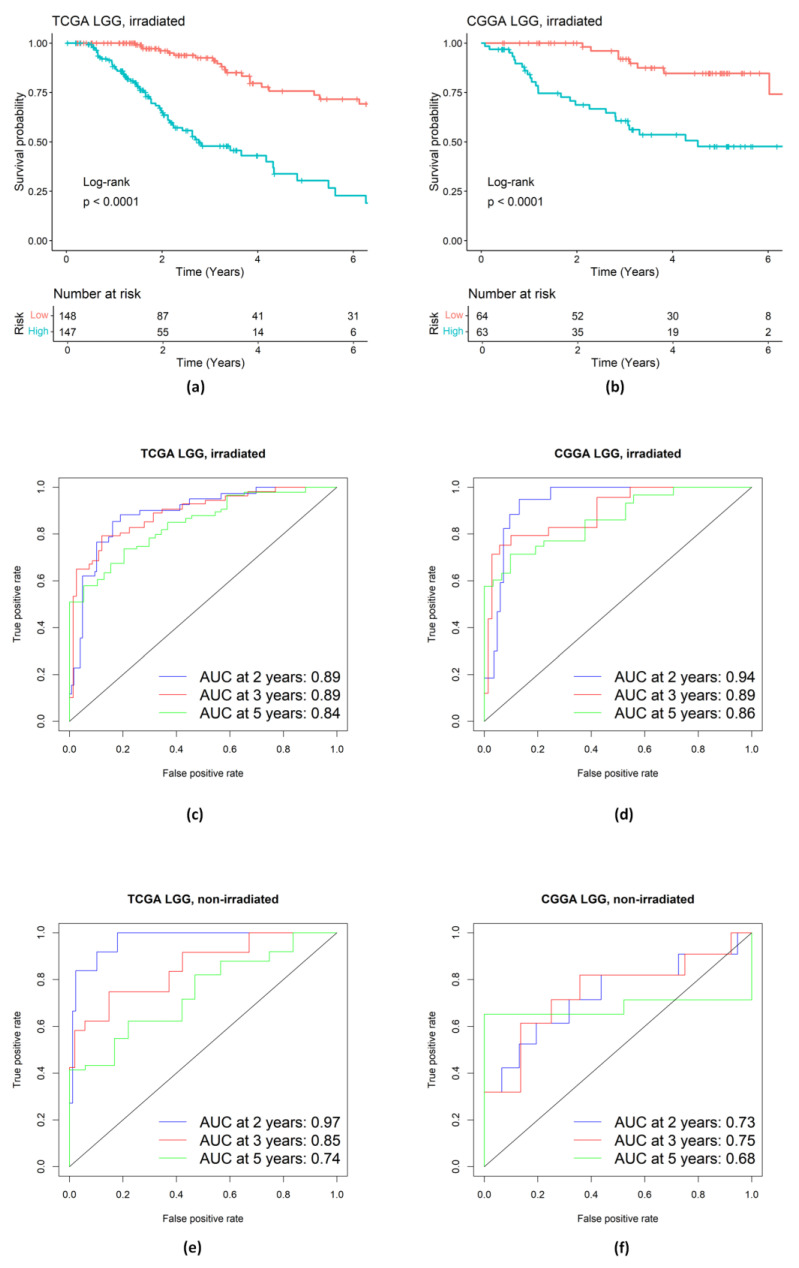
Evaluation of prognostic performance of the integrated genetic signature in the three pathways. (**a**,**b**) KM survival analysis of high- and low-risk groups of LGG patients treated with radiation in the training TCGA dataset and in the validation CGGA dataset. (**c**–**f**) Time-dependent ROC analysis of LGG patients in the training TCGA dataset and in the validation CGGA dataset.

We assessed the performance of this gene signature in LGG patients. The results of KM and time-dependent ROC analyses are provided in Figure 10a–f. From these results, we can see that the identified gene signature from the three pathways shows promising predictive accuracy for irradiated LGG patients with irradiation but not for non-irradiated LGG patients. Also, the three pathways might provide useful targets for developing novel therapies in human gliomas.

## 3. Discussion

Recently, preclinical mechanistic studies suggested that the treatment via combining CSF-1R inhibition (e.g., via BLZ-945) with RT can enhance RT-induced antitumor immunity and lead to long-term improvement in outcomes in murine gliomas. It is of clinical importance to identify key molecular pathways underlying the mechanism of the combination therapy and figure out whether these promising findings from the mouse studies can be successfully translated to gliomas in human patients. In this paper, we identified differentially expressed genes (DEGs) in three significant signaling pathways (PI3K/AKT pathway, Hippo pathway, and Notch pathway). Using key DEGs in the three pathways, we constructed a 4-gene predictive model to investigate resistance to radiotherapy in glioma patients and the advantages of combination therapy. This translational approach borrows the strength from available data in animal models and existing human glioma cohorts. Our Cox model results suggest that CSF-1R inhibition via BLZ-945 with RT has the potential to target the identified pathways to overcome RT resistance. The high AUCs of our identified signature indicate that the models can effectively predict the survival of irradiated LGG patients. For the combined gene signature from the three pathways, as detailed in Section 2.4, the time-dependent area under the curves (AUCs) in the TCGA training set for 2-year, 3-year, and 5-year survival rates were 0.89, 0.89, and 0.84, respectively (Figure 10c). In the testing CGGA dataset, the time-dependent AUCs for 2-year, 3-year, and 5-year survival rates were 0.94, 0.89, and 0.86, respectively (Figure 10d). Notably, this signature did not accurately predict the survival in non-irradiated LGG patients. For the testing CGGA dataset of non-irradiated patients, the time-dependent AUCs for 2-year, 3-year, and 5-year survival rates were 0.73, 0.75, and 0.68, respectively (Figure 10f). Similar results were obtained for the gene signature within each pathway (Figure 4f, Figure 6f and Figure 8f). This might indicate that the gene signature for irradiated patients does not work for non-irradiated patients and may be related to the fact that animal studies indicated limited survival advantages of CSF-1R inhibition alone in mouse glioma models [3,4,10]. From these mouse data and Cox model results, it is reasonable to expect that the combination of radiotherapy and CSF-1R inhibition therapy can improve survival in human glioma patients. In particular, our Cox regression models and ROCs indicate that high expression levels of the signature genes can accurately predict the short survival of the RT-treated LGG patients. Furthermore, our results shed light on the mechanism of the CSF-1R inhibition to mitigate resistance to RT. Radiotherapy increases the release of CSF-1 from tumor cells and attracts macrophages to TME [6]. The binding of CSF-1 to CSF-1R in macrophages can activate PI3K/AKT, Hippo, and Notch signaling pathways, which polarize macrophages to the pro-tumor M2 phenotype [1]. The pro-tumor M2 phenotype macrophages can help tumor cells escape immune surveillance. Inhibition of CSF-1R can downregulate the three pathways (Figure 3b, Figure 5b, Figure 7b and Figure 9b), which will reduce the number of macrophages polarized to the pro-tumor M2 phenotype as observed in Akkari et al. (2020), and thus improve the survival of glioma mice [10]. One might likely use CSF-1R inhibition (e.g., via BLZ-945) in combination with RT to lower expression levels of these signature genes and prolong the survival of the LGG patients. Therefore, the three identified pathways and key molecules might serve as druggable targets via BLZ-945 to overcome the RT resistance in human patients.

The three significantly enriched pathways and the related genes that we identified yielded insights into the molecular mechanism of the survival advantages of glioma mice and human LGG glioma patients using combination therapy over RT-only therapy. Each of the pathways and related genes has previously been extensively reported in the existing literature. The results of Akkari et al. (2020) [10] suggested that the TGF-β/Hippo and Notch signaling pathways were elevated in TAMs at recurrent glioma mice, including the gene NOTCH4. However, we found that NOTCH4 was not significantly associated with patient survival in TCGA. Various studies have shown that the activation of the PI3K/AKT signaling pathway is significantly associated with resistance to CSR-1R inhibition, radiotherapy, and other therapies [3,12]. Therefore, downregulation of the PI3K/AKT pathway is likely to overcome the drug resistance of RT and CSF-1R inhibition. A study demonstrated that Yes-associated protein 1 (YAP1) promotes the metastasis of U251 glioma cells by upregulating Jagged-1 (JAG1) expression and activating the Notch signal pathway [17]. JAG1 was significantly downregulated in the RT plus CSF-1R inhibition group compared with the recurrent RT-treated mice. High levels of JAG1 and NOTCH1/DLL1 were significantly positively associated with short survival in the TCGA cohort, which was consistent with findings in literature [18,19]. Importantly, it has been shown that [18], Notch1 signaling activity was elevated in GBM tissues, and downregulation of the Notch1 pathway by shRNA and MK0752 significantly inhibited the PI3K/AKT/mTOR signaling pathway and weakened the self-renewal, invasion, and tumor growth ability of glioma initiating cells. This is one of the potential mechanisms underlying the combination therapy (RT plus CSF-1R inhibition) that seemingly mitigated resistance to RT-only [12] and CSF-1R inhibition-only therapy [3]. Additionally, experimental results demonstrate that silencing JAG1 yielded a significant decrease in tumor cell proliferation in LGG cell lines, and JAG1 potentially influences PD-L1 in LGG by regulating the PI3K/AKT signaling pathway [19]. The critical role of the Hippo pathway has been investigated in GBM, and the results suggested that the activation of YAP1/WWTR1 was associated with poor prognosis in GBM [20]. It has been found that [21], ITGB8 (β8 integrin) expression was elevated in GBM stem cells and positively associated with stem cell markers in glioma tissues, and could be induced by hypoxia and p38 activation. DCHS1 (dachsous cadherin-related 1) may belong to some canonical cancer molecular pathways in gliomas [22] and has been selected as a marker gene in glioma prognostic signature [23]. Additionally, TGFB2 (Transforming Growth Factor Beta 2) has been identified as a predictor of poor treatment outcomes in pediatric diffuse intrinsic pontine glioma [24].

While early-phase clinical trials can commence solely based on data obtained from animal studies, they are often conducted with constrained sample sizes and limited statistical power, making them easy to produce false-negative results, especially if the wrong class of patients is recruited for the study. Also, findings from animal studies often fail to be translated to human patients due to heterogeneity in human patients and other reasons. Our study indicates the plausibility of the molecular mechanism of the combination therapy to be successfully translated to human LGG patients, which is valuable information for designing targeted clinical trials for gliomas.

Several limitations are associated with the utilization of publicly available data in this study. First, the sample sizes for mouse data and irradiated LGG patients are considerably smaller than what might be optimal, even for preliminary analysis aimed at designing new studies and clinical trials. Second, this study was conducted in human patients using gene expression data from bulk tissue instead of single-cell gene expression. Once the single-cell gene expression data of human is available, one can investigate and translate the radiation resistance mechanism from mice to human with respect to a specific cell type, e.g., specific types of macrophages, which might further improve the efficiency of our method and the interpretability of the findings [1,13]. Thirdly, our study focuses on TAMs, which did not involve their communications with the TCs. The inter-cellular communication plays a substantial role in promoting the progression of low-grade gliomas (LGG) [25]. Fourthly, the TCGA and CGGA clinical datasets were collected many years ago and used a WHO grade classification system, which is outdated and not necessarily identical to the most recent WHO grade definitions. There are also slightly different definitions of low versus high-grade gliomas in the literature. The LGG gliomas we studied in this paper include WHO grade II/III gliomas, as described previously [25,26]. Additionally, due to the high heterogeneity of GBM, the small overall sample size for GBM patients in TCGA, and very few subjects treated with radiation in the TCGA dataset, we did not build a predictive model for the GBM. We expect that the mechanisms of radiotherapy resistance may differ between LGGs and some grade IV gliomas (GBMs) since the GBMs are more heterogeneous than LGGs. Indeed, an existing translational study [26] found that the drug-resistant signature identified in the GBM-proneural subtype also has good prognostic power in LGG. Importantly, our translational approach can be easily applied to GBMs. Future studies might focus on GBM when larger sample sizes are available.

## 4. Materials and Methods

### 4.1. Data Collection of Human Glioma Patients from Publicly Available Database

Two independent and large human glioma cohorts, The Cancer Genome Atlas (TCGA) database (https://cancergenome.nih.gov/) and Chinese Glioma Genome Atlas (CGGA) database (http://www.cgga.org.cn/), were utilized for the translation, signature construction and prediction of the radiation-resistant signature in human. A set of 478 LGG subjects in TCGA and a set of 163 LGG subjects in CGGA, which have matched clinical information and gene expression profiles (RNA-seq data), were collected. Our objective is to identify key genes and molecular pathways underlying the resistance to radiotherapy and the observed efficacy of the combination therapies in preclinical studies and to translate the identified mechanism from mouse to human. The gene signature was constructed among the irradiated LGG patients who had been treated with radiotherapy in both databases (TCGA: n = 295; CGGA: n = 127). We also examined the selected genes’ performance in non-irradiated LGG patients (TCGA: n = 183; CGGA: n = 36). TCGA was used as the training set for signature identification, model construction, and performance evaluation, while CGGA was used as the testing set to validate the predictive performance. Moreover, the corresponding clinical characteristics, including age, sex, and grade of our cohorts, were provided in Table 1.

### 4.2. Differentially Expressed Gene (DEG) Analysis Using Preclinical Mouse Trials

To gain insight into how CSF-1R inhibition improves survival in response to radiation resistance in mice, we started by identifying differentially expressed genes (DEGs) between the monotherapy (RT-only) and combination treatment groups with four mouse samples in each group. The identification was conducted separately in macrophages (MDM) and microglia (MG), both of which were tumor-associated macrophages (TAMs) with different developmental origins [27,28]. DEGs between different treatment groups (monotherapy of radiation versus combination treatment of radiation plus CSF-1R inhibition) were identified using DESeq2 [16]. The significance thresholds were set as *p*-value < 0.05 and log2FoldChange > 0.5 using the Wald test and mean fit type in DESeq2. Subsequently, we combined the DEGs in MDM and MG together and translated the DEGs identified from mouse samples into those in the human genome based on the orthology mapping package (Orthology.eg.db, version 3.18.0). Only those DEGs that have orthology in both human and mouse were used for further analysis.

### 4.3. Evaluation of Predictive Performance of the Identified Gene Signature

The log-rank test and time-dependent ROC were used to evaluate the predictive performance of the identified gene signature. Using gene expression profile X, we first calculated risk scores $f\left(X\right)=X^{\prime }\hat{\beta }$ for LGG patients received radiotherapy in both training and validation datasets, in which $\hat{\beta }$ denoted regression coefficients (log hazard ratio) derived from a multivariate Cox regression model in the training set. Then, glioma patients were classified into high-risk or low-risk groups by choosing the median of risk scores as a cutoff in each dataset, indicating poor or good prognoses, respectively. Kaplan–Meier (KM) curves were generated to summarize patient survival in distinct risk groups, and a log-rank test was conducted to assess whether survival curves for high-risk and low-risk groups were significantly different. Time-dependent ROC analysis was performed to evaluate the accuracy of the identified signature in predicting 2-year, 3-year, and 5-year survival rates. A larger value of the area under the ROC curve (AUC) indicates better predictive power of the gene signature.

## Figures and Tables

**Table 1:** Summary of patient demographics and clinical characteristics used in the Cox models.

		Age		Sex		Grade	
		Median	Range	Male	Female	II	III
TCGA (irradiated)		43	14–87	160	135	94	201
TCGA (non-irradiated)		39	17–75	99	84	138	45
CGGA (irradiated)		40	10–75	82	45	83	44
CGGA (non-irradiated)		38.5	18–74	19	17	19	17

## Data Availability

The mouse RNA-seq gene expression data are available on the Gene Expression Omnibus (GEO) website (https://www.ncbi.nlm.nih.gov/geo/ (accessed on 30 May 2023)) under the accession number GSE99537. TCGA glioma data can be downloaded from The Cancer Genome Atlas (TCGA) database (https://cancergenome.nih.gov/). CGGA glioma data can be downloaded from the Chinese Glioma Genome Atlas (CGGA) database (http://www.cgga.org.cn/).

## Supplementary Materials

The following supporting information can be downloaded at https://www.mdpi.com/article/10.3390/ijms25053076/s1.
