# Supplementary material for: Identification of Key Molecular Pathways and Associated Genes as Targets to Overcome Radiotherapy Resistance Using a Combination of Radiotherapy and Immunotherapy in Glioma Patients
Source: Int J Mol Sci. 2024 Mar 6;25(5):3076. doi: 10.3390/ijms25053076 (PMC10931693; doi:10.3390/ijms25053076)
Supplement: Supplementary file 1 [file ijms-25-03076-s001.zip › ijms-2877755-supplementary.pdf]

Firstly, we list the associated genes for each of the 3 pathways in Table. S1.

Table S1. The list of available enrichment analysis genes for each signaling pathway.

**PI3K/AKT pathway:** IRS1, PPP2R5A, LAMA4, SOS2, AKT1, ITGB8, THBS4, NOS3, RET, COL4A1, PPP2CB, TNR, GNG3, TNC, ANGPT2, COL4A2, PPP2R2B, COL9A3, PDGFRA, COL1A1, EFNA1, IFNB1, COL6A1, COL1A2, GNG11, MAGI2, PPP2R2C, NTRK2, COL6A2, ITGA7, EIF4E, IGFBP3, PXDN, CNTFR, PIK3CA, PIK3CB, PTEN, ACTB, TSC1, MTOR, RPS6KB1, EIF4EBP1
**Hippo pathway:** TGFB2, WWC1, DLG4, WNT5A, FZD3, AXIN1, SMAD4, BMP7, SOX2, MOB1B, PPP2CB, BMP5, WNT7B, PPP2R2B, WNT7A, CTNNA2, PPP2R2C, PARD3, YAP1, DCHS1, WWTR1
**Notch pathway:** DLL1, TLE4, HEYL, ATXN1L, NOTCH4, DLL3, RBPJL, NOTCH1, HES1, JAG1, JAG2

We have performed the Kaplan-Meier survival analysis for each of the genes we identified from the three pathways in Section 2. Also, we provided the barplots that showing the expression of each selected genes in microglia (MG) in different treatment groups of mice. The results are presented in Fig. S1. These results suggested that our identified genes are significantly assoicated with the survival of the irradiated LGG patients. More importantly, down-regualting these iendetified genes and their associated pathways is likely to overcome the RT-resistance for LGG patients.

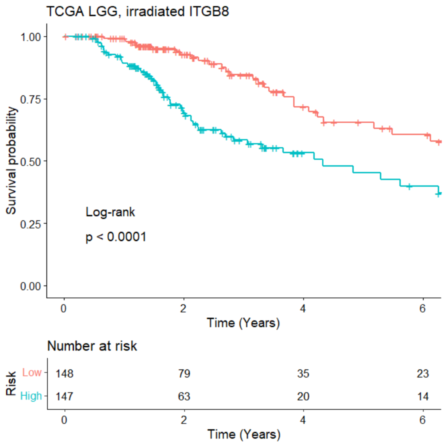

(1a)

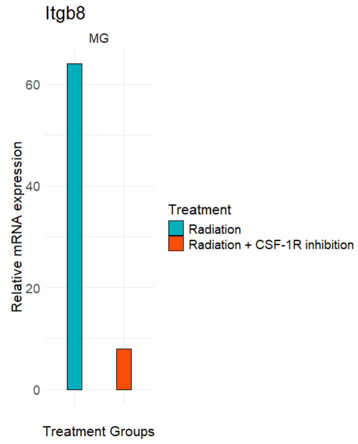

(1b)

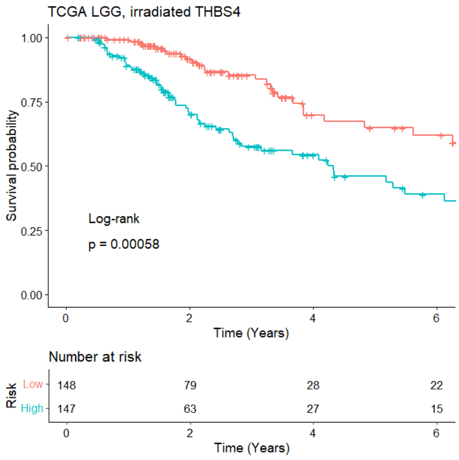

(2a)

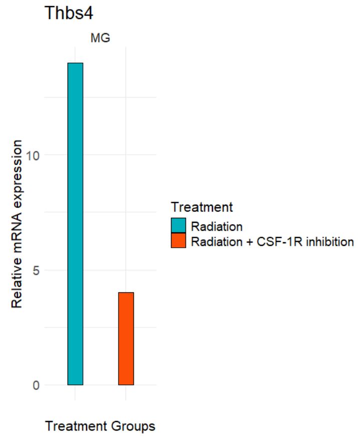

(2b)

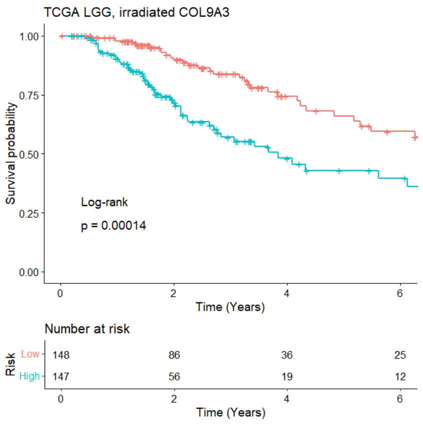

(3a)

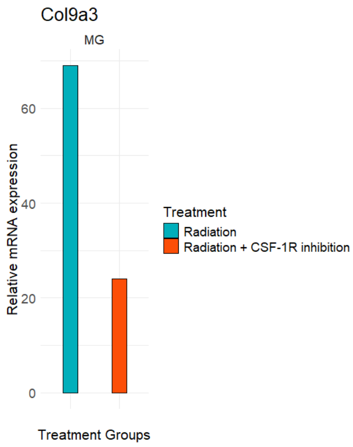

(3b)

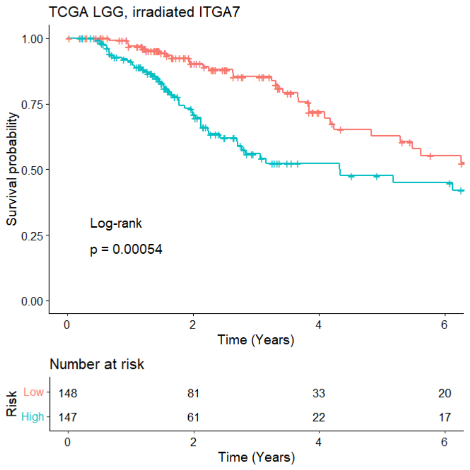

(4a)

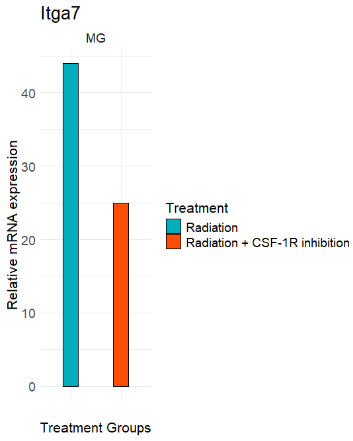

(4b)

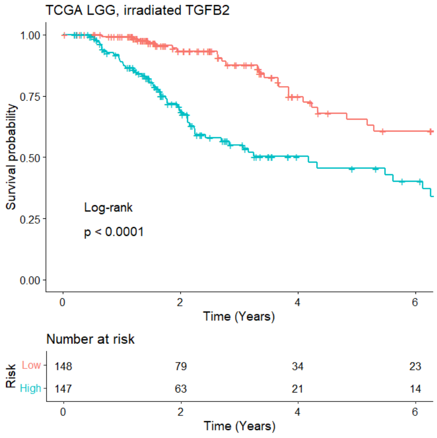

(5a)

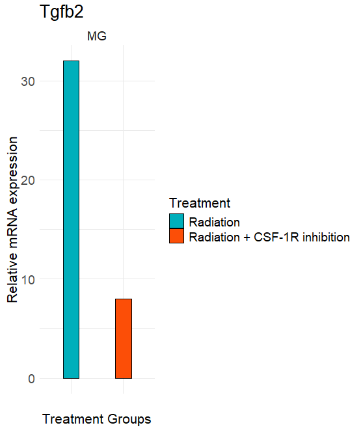

(5b)

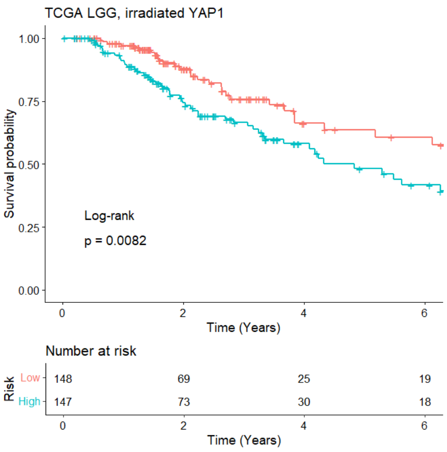

(6a)

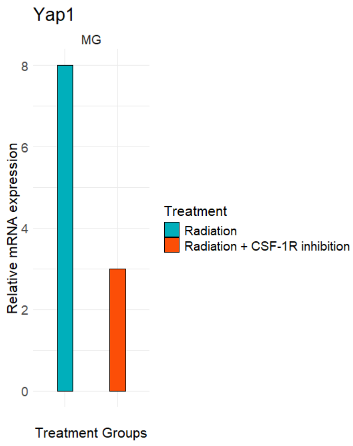

(6b)

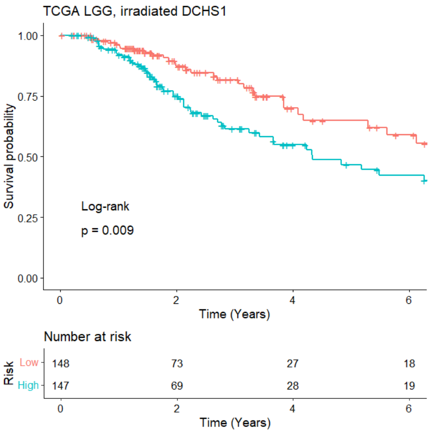

(7a)

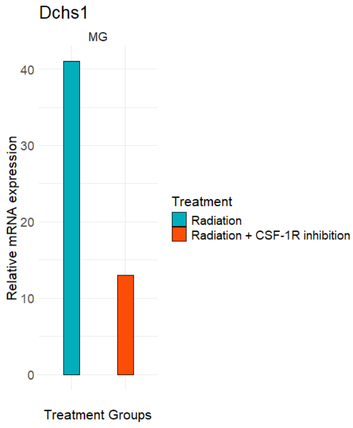

(7b)

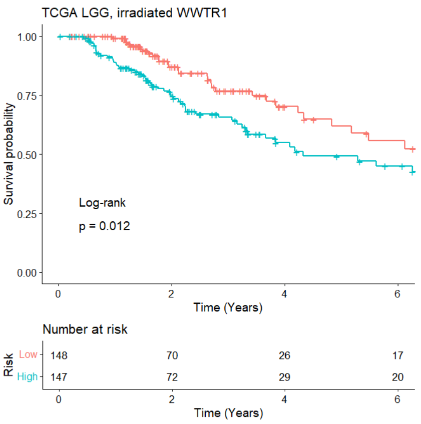

(8a)

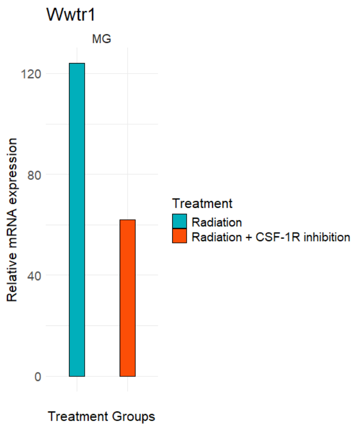

(8b)

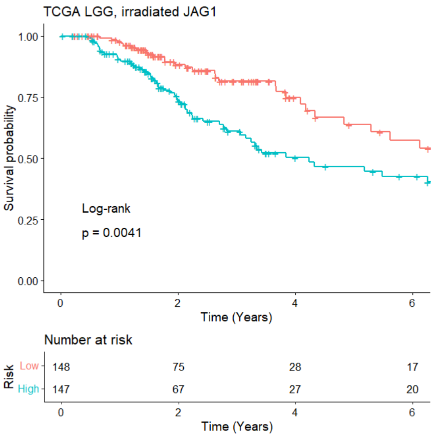

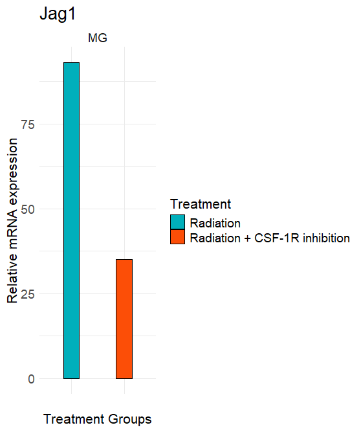

(9a)

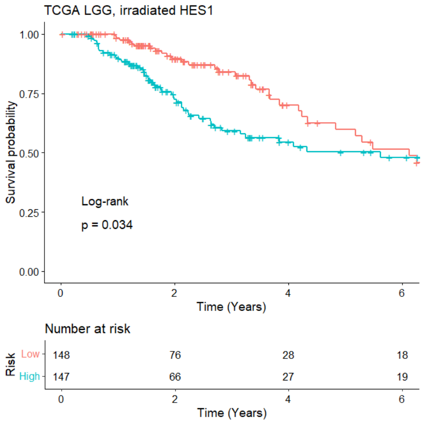

(9b)

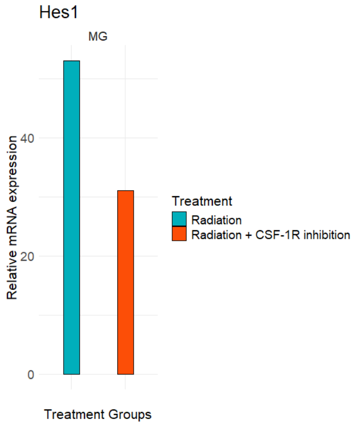

(10a)

(10b)

Figure S1. KM survival analysis of high- and low-risk groups of LGG patients treated with radiation in the training TCGA dataset for each selected gene (1a-10a). Barplot representing the expression of each selected genes in MG in different treatment groups of mice (1b-10b).
